# The novel function of tumor protein D54 in regulating pyruvate dehydrogenase and metformin cytotoxicity in breast cancer

**DOI:** 10.1186/s40170-018-0193-4

**Published:** 2019-01-24

**Authors:** Yongxian Zhuang, Reynold C. Ly, Carleigh V. Frazier, Jia Yu, Sisi Qin, Xiao-Yang Fan, Matthew P. Goetz, Judy C. Boughey, Richard Weinshilboum, Liewei Wang

**Affiliations:** 1 Division of Clinical Pharmacology, Department of Molecular Pharmacology and Experimental Therapeutics, Mayo Clinic, 200 First Street SW, Rochester, MN 55905 USA; 2 Division of Clinical Pharmacology, Department of Molecular Pharmacology and Experimental Therapeutics, Mayo Clinic Graduate School of the Biomedical Sciences, Rochester, MN 55905 USA; 3 Department of Biology, Fisk University, Nashville, TN 37208 USA; 4 Department of Oncology, Mayo Clinic, Rochester, MN 55905 USA; 5 Department of Surgery, Mayo Clinic, Rochester, MN 55905 USA

**Keywords:** Tumor protein D54 (TPD54), Pyruvate dehydrogenase (PDH), Pyruvate dehydrogenase kinase (PDK), Metformin, Mitochondrial reactive oxygen species (ROS)

## Abstract

**Background:**

The role of tumor protein D54 in breast cancer has not been studied and its function in breast cancer remains unclear. In our previous pharmacogenomic studies using lymphoblastoid cell line (LCL), this protein has been identified to affect metformin response. Although metformin has been widely studied as a prophylactic and chemotherapeutic drug, there is still a lack of biomarkers predicting the response to metformin in breast cancer. In this study, we revealed the novel function of TPD54 in breast cancer through understanding how TPD54 altered the cancer cell sensitivity to metformin.

**Methods:**

The role of TPD54 in altering cellular sensitivity to metformin treatment was carried out by either knockdown or overexpression of TPD54, followed by measuring cell viability and reactive oxygen species (ROS) production in MCF7 breast cancer cell line and breast cancer patient-derived xenografts. Functional analysis of TPD54 in breast cancer cells was demonstrated by studying TPD54 protein localization and identification of potential binding partners of TPD54 through immunoprecipitation followed by mass spectrometry. The effect of TPD54 on pyruvate dehydrogenase (PDH) protein regulation was demonstrated by western blot, immunoprecipitation, and site-directed mutagenesis.

**Results:**

TPD54 inhibited colony formation and enhanced cellular sensitivity to metformin treatment in MCF7 cells and breast cancer patient-derived xenografts. Mechanistic study indicated that TPD54 had mitochondrial localization, bound to and stabilized pyruvate dehydrogenase E1α by blocking pyruvate dehydrogenase kinase 1 (PDK1)-mediated serine 232 phosphorylation. TPD54 knockdown increased PDH E1α protein degradation and led to decreased PDH enzyme activity, which reduced mitochondrial oxygen consumption and reactive oxygen species (ROS) production, thus contributing to the resistance of breast cancer cells to metformin treatment.

**Conclusion:**

We have discovered a novel mechanism by which TPD54 regulates pyruvate dehydrogenase and affects the sensitivity of breast cancer to metformin treatment. Our findings highlight the important post-translational regulation of PDK1 on PDH E1α and the potential application of TPD54 as a biomarker for selecting tumors that may be sensitive to metformin therapy. These provide new insights into understanding the regulation of PDH complexes and the resistance mechanisms of cancer cells to metformin treatment.

**Electronic supplementary material:**

The online version of this article (10.1186/s40170-018-0193-4) contains supplementary material, which is available to authorized users.

## Background

TPD54 is a tumor protein which shares functional characteristics with tumor protein D52 (TPD52) [1, 2, 3]. TPD52 has been reported to be amplified in breast, ovarian, and prostate cancers and has also been associated with poor prognosis [4–7]. Its known functions involve the regulation of cell proliferation and migration [8–10]. Recent studies have also shown that TPD54 affects cell proliferation, cell adhesion, and invasion [11]. Furthermore, TPD54 has been shown to have opposite effects to TPD52 in oral squamous cell carcinoma (OSCC) by preventing colony formation and cell migration [12]. However, the underlying mechanisms of TPD54’s role in cancer, including breast cancer, remain unclear. Even less information is available with regard to its role in treatment response.

We previously performed a pharmacogenomics study using lymphoblastoid cell lines (LCLs) to identify genes that might contribute to metformin response that included attempts to identify metformin response biomarkers and potential new regulators of pathways involved in metformin response ([13, 14]. One of the genes whose expression level was highly associated with metformin IC50 in LCLs was the tumor protein D54 (TPD54) (*p* value, 1.11 × 10^−4^).

One identified mechanism of metformin’s anticancer action is through the increased production of mitochondrial reactive oxygen species (ROS) by inhibition of mitochondrial complex I [15–18]. It has been shown that pyruvate dehydrogenase (PDH) activity can significantly affect reactive oxygen species production and cellular sensitivity to metformin associated with mitochondrial oxidative phosphorylation [19]. The observation that tumor protein TPD54 alters the cellular sensitivity to metformin treatment leads us to hypothesize that TPD54 might be involved in the regulation of PDH related mitochondrial function and cancer metabolism.

Cancer cells are known to have metabolic alterations with higher glucose consumption and reduced oxidative phosphorylation in the mitochondria even under normoxic conditions to support the anabolic requirements for cell growth and proliferation [20]. Pyruvate dehydrogenase (PDH) is the key enzyme linking glycolysis and tricyclic acid cycle (TCA) [21–25]. Emerging evidences suggest that cancer metabolic alterations may in part result from the inhibition of pyruvate dehydrogenase complex [23, 26, 27]. PDH complex activity is mainly controlled by phosphorylation and dephosphorylation of the PDH E1α subunit at three different serine sites (S293, S300, and S232). Phosphorylation of PDH E1α at serine 293 by PDK2 is the most well-known mechanism for PDH E1α enzyme inactivation. The role of phosphorylation at serine 232 and serine 300 in enzyme inactivation is not well understood [28]. Four pyruvate dehydrogenase kinases (PDK1, PDK2, PDK3, and PDK4) have been identified in mammalian cells which have varied catalytic activity toward PDH E1α. To date, only PDK1 is known to phosphorylate PDH E1α at serine 232, but its role in the regulation of enzyme activity is not well understood.

In this study, we identified the interaction between TPD54 and PDH by examining how TPD54 affected cell sensitivity to metformin and further revealed that TPD54 stabilized PDH E1α protein by preventing PDK1-mediated phosphorylation. These findings will provide novel insights in understanding the role of TPD54 in the regulation of PDH complex, cancer metabolic reprogramming, and the mechanisms of cancer resistance to metformin treatment.

## Methods

### Cell lines

The breast cancer cell lines, MCF-7, T47D, BT549, and MDA-MB-231, were purchased from ATCC and maintained in DMEM media containing 10% fetal bovine serum supplemented with 1× Gibco Antibiotic-Antimycotic.

### Sytox green staining for live and dead cells

Cells were plated on 96-well plates and grown to 70% confluency. Cells were treated as indicated, followed by the addition of SYTOX® green nucleic acid stain (10 μM), and were then incubated for an additional 20 min before being read on a fluorescence plate reader at excitation/emission wavelengths of 485/535 nm with a 515 nm cutoff. Cells were then permeabilized with Triton X-100 (0.4%) for 30 min, followed by a second reading to determine the total level of DNA staining, a surrogate for total cell number.

### CyQUANT direct cell proliferation assay

Cells were plated on 96-well plates and grown to 70% confluency. After cells were treated as indicated, CyQUANT 2× detection reagent was prepared and added directly to the cells in complete medium and were incubated for 30 min. Fluorescence intensities were measured with a fluorescence microplate reader at the excitation/emission wavelengths of 480/535 nm. Mean fluorescence intensity (MFI) was plotted to represent live cells.

### Western blots and antibodies

Cells were grown in 35 mm dishes and harvested with 1× SDS sample buffer following procedures described in previous publications [29]. Briefly, proteins were separated in Biorad precast polyacrylamide gels and transferred onto membranes using the Biorad ready-to-assemble transfer kit. PVDF membranes were blocked with 5% milk in 1× TBST for 1 h and incubated with the primary antibody overnight at 4 °C. Following this incubation, membranes were washed in 1× TBST for 30 min, followed by incubation with secondary antibody for an additional hour. Proteins were detected by SuperSignal™ West Dura Extended Duration Substrate (Cat#34075) using the ChemiDoc™ Touch Imaging System. The antibodies used were as follows: TPD54 (Abcam, AB194938), Pyruvate dehydrogenase (PDH) WB antibody cocktail (Abcam, ab110416), NDUFB8 antibody [20E9DH10C12] (Abcam, AB110242), PDH E1α (Abcam, ab110334), PDH E1α (Proteintech, 18068-1-AP), PDH E1α (Proteintech, 66,119-1-Ig), PhosphoS232 PDH E1α (LSBio, LS-C265964), PhosphoS293 PDH E1α (Novus, NB110-93479), phospho-Histone H2A.X (Ser139) (20E3) Rabbit mAb (Cell signaling, Cat#9718), vinculin (Cell signaling, Cat#4650), Actin (Cell signaling, cat# 3700), Cleaved PARP (Cell signaling, Cat#5625), ubiquitin (Abcam, ab7780), PDK1 (Santa Cruz,sc36203), PDK2 (Santa Cruz, sc517284), SNAP-Tag (New England Biolab, p9310s), mCherry (Sigma, SAB2702291), Peroxidase-AffiniPure F(ab′)2 Fragment Goat Anti-Rabbit IgG, Fc Fragment Specific (Jackson ImmunoResearch, 111-036-046), Peroxidase-AffiniPure F(ab′)2 Fragment Goat Anti-Mouse IgG, Fc Fragment Specific (Jackson ImmunoResearch, 115-036-071), Peroxidase-IgG Fraction Monoclonal Mouse Anti-Rabbit IgG, Light Chain Specific (Jackson ImmunoResearch, 211-032-171), and Peroxidase-AffiniPure Goat Anti-Mouse IgG, Light Chain Specific (Jackson ImmunoResearch, 115-035-174). Additional antibodies included Goat Anti-Mouse IgG (H + L) (Alexa Fluor 594) (Abcam, ab150116), Goat Anti-Rabbit IgG (H + L) (Alexa Fluor 488) (Abcam, ab150077), Anti-rabbit IgG, HRP-linked antibody (Sigma, 7074S), and Anti-mouse IgG HRP-linked antibody (Sigma, 7076S).

### TPD54 knockdown and overexpression

Specific siGENOME siRNA SMARTpool® reagents against TPD54 as well as a negative control, siGENOME Non-Targeting siRNA, were purchased from Dharmacon Inc. (Lafayette, CO, USA). Detailed sequence information was provided in Additional file 1: Supplementary Materials and Methods. MCF-7 cells were transiently transfected with negative control or TPD54 siRNAs for 24 h using Lipofectamine RNAimax. Cells were then treated with metformin at different concentrations for 48 h.

shRNA plasmids (shTRC2 control and shTPD54 shRNA plasmids) were purchased from Sigma-Aldrich. MCF-7 cells were transfected with shRNA plasmids using the Lipofectamine 2000 transfection reagents. Transfected cells were then incubated in the presence of puromycin at 2 μg/mL for 2 weeks to generate stably transfected cells. Single colonies with low TPD54 expression were then selected for further downstream experiments.

TPD54 (NM_199359.2) was overexpressed in MCF-7 cells and patient-derived xenografts using lipofectamine 2000. Cells were treated as indicated after transfection for 1 day.

### MitoSox staining

Cells were treated as indicated for 2 days and were then stained with MitoSOX (5 μM) (Molecular probe, M36008) for 15 min. Cells were washed and trypsinized, followed by the addition of 1 ml DMEM/10% FBS media to the cells. MitoSOX levels in an equal number of cells per treatment condition were measured using a microplate reader (Tecan Infinity M1000) that was set to 510 nm excitation (Ex bandwidth:10 nm) and 595 nm emission (Em bandwidth: 35 nm) wavelengths.

### Immunofluorescence

Cells were plated in 16-well cover-glass slips (Thermo Fisher, C37000) and were transfected with dsMito for mitochondria labeling. They were then treated with the indicated drug for 2 days and were washed with 1× PBS before being fixed with 4% paraformaldehyde for 10 min, followed by permeabilization using 0.1% Triton X-100. Cells were then blocked with 3% BSA for 30 min and were incubated with specific primary antibody overnight at 4 °C. They were then incubated with the appropriate Alexa Fluor antibody for 1 h at room temperature. The cells were stained with DAPI before visualization using Zeiss LSM 780 Confocal Microscope at × 100 magnification. Overlap coefficient was quantified using Zen software and presented in Additional file 2: Figure S2.

### Mitochondria/Cytosol Fractionation preparation

MCF-7 cells were cultured in 100-mm dishes, washed with 1× PBS and pelleted by centrifugation at 1500 rpm for 5 min. A Mitochondria/Cytosol Fractionation Kit (K256-25, BioVision) was used to isolate cellular mitochondria and cytosol.

### Immunoprecipitation

Cell extracts were prepared using a lysis buffer described previously [29]. Briefly, extracts were sonicated for 10 s and then centrifuged at 14000 rpm at 4 °C for 15 min. Equal amounts of total protein from each extract were incubated with 2 μg of TPD54 (Abcam, AB194938) or PDH E1α antibodies (Proteintech, 66119-1-Ig) overnight at 4 °C on a rotator. Protein A-conjugated agarose beads (Pierce™ Protein A Agarose, 20,333) (30 μl) were then added and incubated for 1 h on a rotator at 4 °C. The beads were pelleted at 4000 rpm at 4 °C and then were washed five times in 500-μl lysis buffer. Precipitated proteins were then dissolved in SDS-sample buffer and separated by SDS-PAGE for western blotting.

### Colony formation assay

MCF-7 cells were initially plated at 1000 cells per 60-mm dish for 24 h. They were then treated with metformin as indicated and were incubated for 12 days at 37 °C/5% CO_2_. Following the 12-day treatment, the cells were washed with 1× PBS and fixed with 70% ethanol for 5 min. Colonies were then stained with Coomassie Blue (40% methanol, 12% glacial acetic acid, 0.24% Coomassie Blue), washed by 1× PBS, and imaged using ChemiDoc™ Touch Imaging System.

### Oxygen consumption rate

Oxygen consumption rate (OCR) was measured using a Seahorse XFp analyzer according to the manufacturer’s instructions (Agilent, USA). Cells were plated at 15,000 cells per well in XFp miniplate for 24 h. They were then washed and equilibrated in buffer free media for 30 min at 37 °C in a CO_2_-free incubator before transferring to the XFp analyzer. OCR was measured every 30 min.

### Pyruvate dehydrogenase (PDH) activity colorimetric assay

Cells were cultured in 100-mm dishes for 24 h and then were treated with metformin as indicated for 24 h. They were then trypsinized and counted using a cell counter. One million cells per sample were pelleted by centrifugation at 1500 rpm for 5 min. Cells were then lysed using the Pyruvate Dehydrogenase (PDH) Activity Colorimetric Assay Kit following the manufacturer’s instructions (K679-100, Biovision). Kinetic PDH activity was read every 10 min during a total time of 60–90 min. PDH kinetic curves were then plotted.

### PDK1 and PDK2 knockdown

siRNA-targeting PDK1 was purchased from Santa Cruz (sc36203). Specific siGENOME siRNA SMARTpool® reagents against PDK2 were purchased from Dharmacon Inc. (Lafayette, CO, USA). Briefly, cells were transiently transfected with negative control or PDK1 and PDK2 siRNAs for 24 h using Lipofectamine RNAimax. The cells were then treated with metformin at different concentrations for 48 h. Detailed sequence information was provided in Additional file 1: Supplementary Materials and Methods.

### PDH E1α site-directed mutagenesis

A PDH E1α plasmid was purchased from Addgene (Plasmid #58195). Site-directed mutagenesis was performed with the QuikChange II Site-Directed Mutagenesis Kit (Agilent, USA) to prepare mutants PDH E1α S232A, PDH E1α S293G, and PDH E1α S330A. Detailed primer information and sequencing results are provided in the supplementary materials (Additional file 3).

### 3D culture of breast cancer patient-derived xenospheroids

Human breast tumor xenografts were maintained subcutaneously in NSG female mice as described previously [30, 31]. 3D organoids were generated from the PDX tumors as described previously [32]. Specifically, human breast tumor cells were isolated using a tumor dissociation kit (Human) and mouse cell depletion kit purchased from Miltenyi Biotec Inc. Briefly, the tumor was cut into small pieces of 2–4 mm, followed by enzymatic digestion and mechanical dissociation on gentleMACS™ Dissociators at 37 °C for 1 h. Afterwards, samples were applied to a MACS SmartStrainer (70 μm) to remove any remaining large particles from the single-cell suspension. The cell suspension was then centrifuged at 300×g for 7 min to pellet-dissociated cells. Erythrocytes or dead cells were removed using Red Blood Cell Lysis Solution obtained from Miltenyi Biotec Inc. Remaining cells were then incubated with a cocktail of monoclonal antibodies conjugated with MACS® MicroBeads for 15 min at 4 °C to magnetically label any mouse cells. The cell suspension was then loaded onto a MACS column placed in the magnetic field of a MACS separator. The eluent containing the unlabeled human tumor cells was collected in a clean tube. Cells were then counted and resuspended in DMEM media containing 10% FBS supplemented with 1× Gibco Antibiotic-Antimycotic, 1× Gibco Glutamax, 1× Gibco MEM NEAA, 1 mM Gibco sodium pyruvate, and 5-μM ROCK inhibitor (Y27632 Tocris/ R&D). Tumor cells were then plated into 96-well NanoCulture®Plates (NCP) at 10,000 cells per well. 3D organoids were formed and used for further downstream studies after 7 to 14 days.

### 3D viability assay

3D xenospheroids were lysed using the Promega 3D viability assay. After the xenospheroids were treated as indicated with metformin, 100-μl medium was removed from the 96-well plates, followed by the addition of 100 μl of 3D viability assay reagent for a 30 min incubation at 37 °C with shaking. Results were obtained using a luminescence microplate reader (Tecan Infinity M1000).

### Statistics

Statistical tests were performed using GraphPad Prism (GraphPad Software); Student’s *t* test was used to assess the statistical significance of differences between two conditions, whereas one-way ANOVA was utilized when multiple variables were compared. All figures presented are representative experimental data that have been replicated by independent experiments and are reported as the mean ± SD from triplicate data of one experiment (*p < 0.05, **p < 0.01, ***p < 0.001, and ****p < 0.0001).

## Results

### TPD54 affects cellular sensitivity to metformin treatment accompanied by altered ROS production in MCF7 cells and breast cancer patient-derived xenografts

**Fig. 1:**
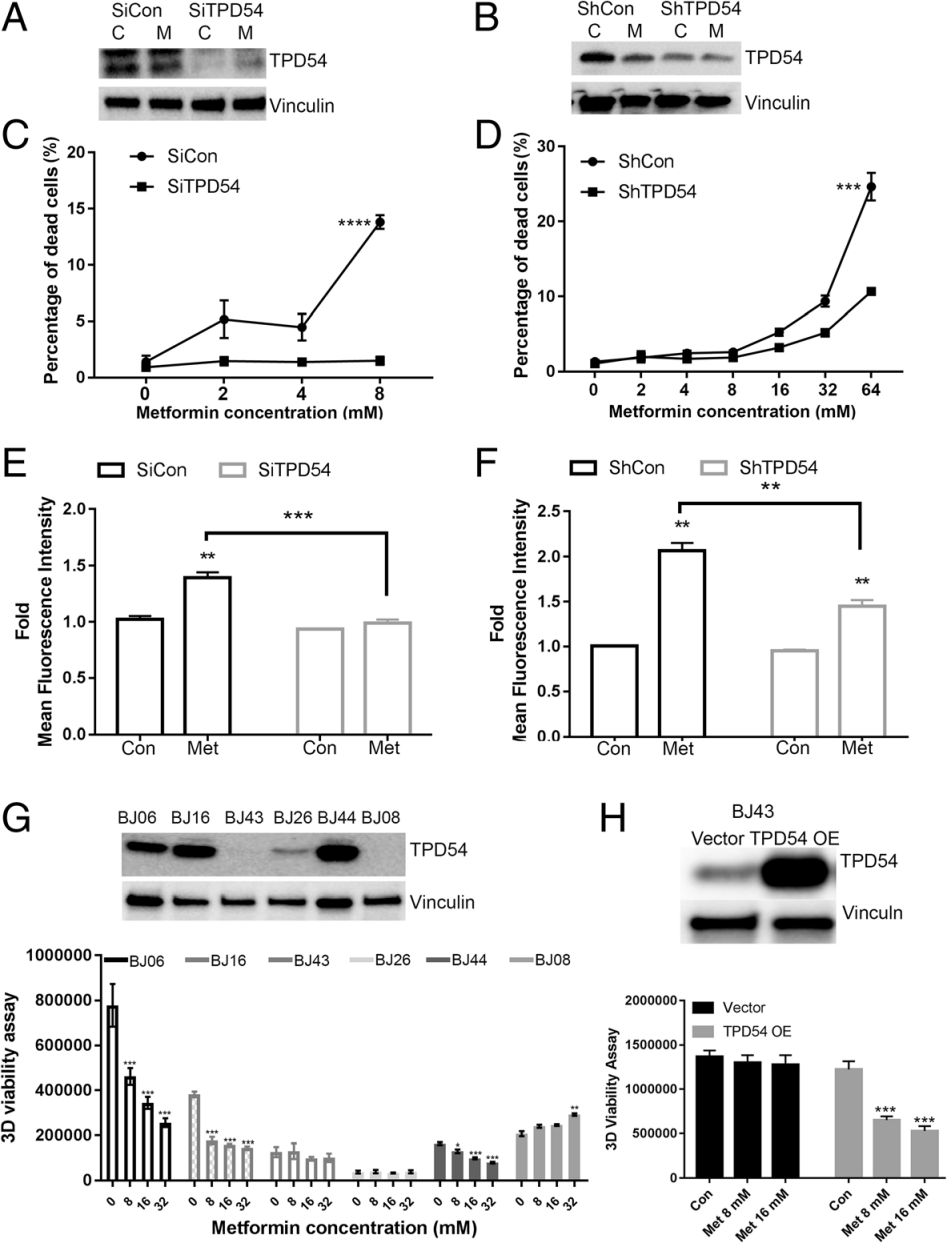
TPD54 affects cellular sensitivity to metformin treatment accompanied by altered ROS production in MCF7 cells and breast cancer patient-derived xenografts. **a** TPD54 protein level was detected by western blot in control and TPD54 transiently knocked down cells treated with or without 8-mM metformin for 2 days. C: 1× PBS, M: metformin **b** TPD54 protein level was detected by western blot in control and TPD54 stably knocked down MCF-7 cells treated with or without 8-mM metformin for 2 days. C: 1× PBS, M: metformin. **c** Percentage of dead cells was plotted in cells transfected with control and TPD54 siRNAs and treated with metformin at indicated concentrations for 2 days. **d** Percentage of dead cells was plotted in control and TPD54 stably knocked down cells treated with metformin at the indicated concentrations for 2 days. **e** Fold changes of Mitosox mean fluorescence intensity representing mitochondrial ROS production was quantified in cells described in **c**. **f** The same assays and quantifications were performed in cells described in **d**. **g** Western blot analysis was performed using lysates from different breast cancer xenospheroids to detect TPD54. 3D cell viability was measured for xenospheroids treated with metformin at the following concentrations for 5 days: 8, 16, and 32 mM metformin. **h** TPD54 was overexpressed in BJ43 and treated with metformin as indicated for 3 days. 3D viability was performed. All data presented are representative experimental data that have been replicated by independent experiments and are reported as the mean ± SD from triplicate data of one experiment. Values are presented as mean ± SD, student *t* test was used to calculate *p* value for two-group comparisons with ****p < 0.0001, ***p < 0.001, **p < 0.01, *p < 0.05)

Our previous finding showed that TPD54 affected metformin IC50 in lymphoblastoid cell lines (LCLs) [13]. We further examined the effects of TPD54 in altering cellular sensitivity to metformin in breast cancer cells by knocking down or overexpressing TPD54. MCF-7 cells were either transiently or stably knocked down using siRNA or shRNA-targeting TPD54 as demonstrated by reduced TPD54 protein with western blot (Fig. 1a, b). Sytox green assay was performed to determine the percentage of dead cells 2 days after the cells were treated with increasing concentrations of metformin. The percentage of dead cells in TPD54 knockdown cells treated with metformin was significantly decreased compared to control cells, most significantly with higher concentration of metformin (Fig. 1c, d), which might be partly due to the fact that high metformin concentrations with short treatment time facilitated the accumulation of metformin into the mitochondria [33]. We also showed similar effects of TPD54 on cell sensitivity to metformin treatment at a low concentration of 2 mM when the cells were treated for more than 4 days (Additional file 4**:** Figure S1A). All supplementary figure legends were presented in Addtional file 5. The rescue experiment through the re-expression of TPD54 in the shTPD54 cell lines was performed which showed that the re-expression of TPD54 increased metformin sensitivity in shTPD54 cells (Additional file 4: Figure S1B and C). To be sure the specificity of the siRNA and shRNA, another pair of control and shRNA-targeting TPD54 was purchased from Santa Cruz to further confirm that the TPD54 knockdown could reduce metformin-induced cell death after treatment for 4 days (Additional file 4: Figure S1D and E). Metformin is known to inhibit mitochondrial complex I. To better understand the effect of TPD54 on mitochondrial function, the control and shTPD54 stable knockdown cells were treated with two other complex I inhibitors (piericidin A or rotenone) for 2 days, and the results demonstrated that the downregulation of TPD54 diminished cell sensitivity to those two complex I inhibitors as observed with metformin (Additional file 4: Figure S1F and S1G). Previously, metformin was shown to increase the production of reactive oxygen species, which contributed to metformin’s mechanism of cell death [34]. This prompted us to compare the levels of mitochondrial reactive oxygen species induced by metformin treatment in control and TPD54 knockdown cells. We demonstrated that metformin could significantly induce ROS production in control cells, but TPD54 knockdown partially blocked the induction of ROS production caused by metformin (Fig. 1e, f). This suggests that TPD54 might be involved in the regulation of ROS production induced by metformin, which in turn, could affect metformin cytotoxicity.

In order to further confirm the correlation between TPD54 and metformin response, next, we examined their relationship using different xenospheroids established from breast cancer patient-derived xenograft tumors [30, 31]. BJ06, BJ16, and BJ43 were triple negative breast cancer, BJ26 and BJ08 were HER2+, and BJ44 were ER+ breast cancer. TPD54 expression levels varied among different breast cancer xenospheroids. All lines with different expression levels of TPD54 were treated with metformin. BJ43, BJ26, and BJ08 had undetectable or low levels of TPD54 compared to BJ06, BJ16, and BJ44. Furthermore, BJ06, BJ16, and BJ44 were relatively sensitive to metformin; BJ43, BJ26, and BJ08 were resistant to metformin treatment even at high concentration of 32 mM (Fig. 1g). Additionally, the overexpression of TPD54 in BJ43 could restore its sensitivity to metformin treatment (Fig. 1h). These results indicate that TPD54 is positively correlated with breast cancer sensitivity to metformin and may be used as a biomarker for predicting metformin response in breast cancer.

### TPD54 interacts with pyruvate dehydrogenase and plays an important role in breast cancer cells

**Fig. 2:**
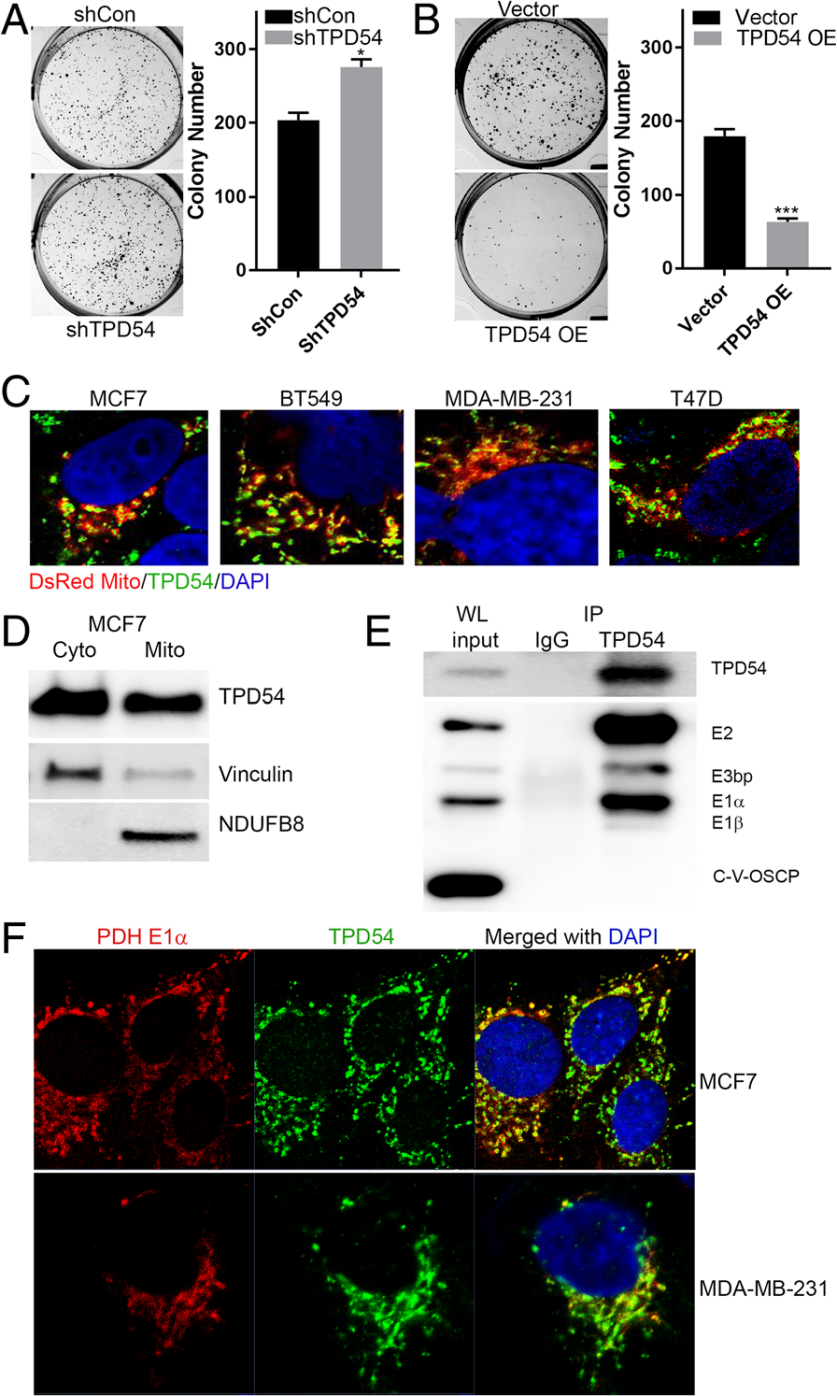
TPD54 interacts with pyruvate dehydrogenase and plays an important role in breast cancer cells. **a** Images and the number of colony formation in control and stable TPD54 knockdown MCF-7 cells are presented. **b** Images and the number of colony formation of vector control and TPD54 overexpressed MCF7 cells are shown**.** Data were reported as the mean ± SD from triplicate data of one experiment. Values are presented as mean ± SD, student *t* test was used to calculate *p* value for two-group comparisons with ***p < 0.001 and *p < 0.05).**c** Endogenous TPD54 protein was detected using immunofluorescence. Mitochondria were labeled by DsRed Mito through transient transfection. The nucleus was labeled by DAPI staining. Images were taken at 200×. **d** Fractionations of cytoplasm (Cyto) and mitochondria (Mito) were performed in MCF-7 cells. Western blots were performed using cytoplasmic and mitochondrial fractions to identify TPD54 subcellular localization. Vinculin and NDUFB8 were used as cytoplasmic and mitochondrial markers and loading controls. **e** Endogenous TPD54 immunoprecipitation was performed in MCF-7 cells. Western blot detected TPD54 pulldown and its associated proteins in PDH complex. WL represents whole cell lysate as input. **f** PDH E1α and TPD54 were detected in MCF7 and MDA-MB-231 cells using immunofluorescence. The nucleus was labeled by DAPI staining. Images were taken at 100×. All data presented were representative experimental data that have been replicated by independent experiments

We have demonstrated that TPD54 plays an important role in affecting cellular sensitivity to metformin treatment in breast cancer cell lines and patient-derived xenografts. However, little is known about its function in breast cancer, even less is known how it affects cell response to drug treatments. Therefore, we investigated its role in influencing cell proliferation and drug response in breast cancer cells. TPD54 inhibited colony formation and cell proliferation in MCF7 cells (Fig. 2a, b, Additional file 6: Figure S2A, B), which further confirmed its biological importance in breast cancer. As we have shown, TPD54 knockdown partially blocked the induction of ROS production caused by metformin (Fig. 1e, f). This suggests that TPD54 is likely to be involved in the regulation of metformin-induced ROS production. Next, confocal microscopy was performed to examine the localization of TPD54 in MCF-7, MDAMB231, BT549, and T47D cells using DsRed Mito to label mitochondria, where the majority of ROS is produced. We found that TPD54 was localized in the cytoplasm as well as mitochondria as demonstrated by images showing its co-localization with dsRed labeled mitochondria (Fig. 2c). The 8-bit grayscale of individual channel and overlap coefficient were presented in Additional file 6: Figure S2C. To further verify TPD54’s mitochondrial localization, we performed western blot analysis using cytoplasmic and mitochondrial fractions. Western blot analysis confirmed that TPD54 had both cytoplasmic and mitochondrial localization in MCF-7 cells (Fig. 2d).

These interesting findings that TPD54 was localized in the mitochondria and involved in the regulation of ROS production supported our hypothesis that TPD54 may play a role in cancer metabolism. Therefore, we immunoprecipitated TPD54 in MCF-7 cells and performed mass spectrometry to identify TPD54 interacting proteins. Two of the top identified proteins that are known to regulate cell metabolisms were pyruvate dehydrogenase A and B subunits (Additional file 6: Mass spectrometry results), suggesting a potential interaction between TPD54 and the pyruvate dehydrogenase (PDH) complex. This interaction was validated by endogenous TPD54 immunoprecipitation performed with MCF-7 cells, in which all subunits of PDH were detected in the immunoprecipitates (Fig. 2e). Confocal microscopy further confirmed that TPD54 interacted with the PDH complex by staining TPD54 and PDH E1α subunit in both MCF7 and MDA-MB-231 cells (Fig. 2f). The 8-bit grayscale of individual channel and overlap coefficient were presented in Additional file 2: Figure S2D. Collectively, these results support that TPD54 interacts with pyruvate dehydrogenase and their interaction might have an important biological role in breast cancer.

### TPD54 is positively correlated with PDHE1 α protein and PDH complex enzyme activity, and through it changes the sensitivity of cells to metformin

**Fig. 3:**
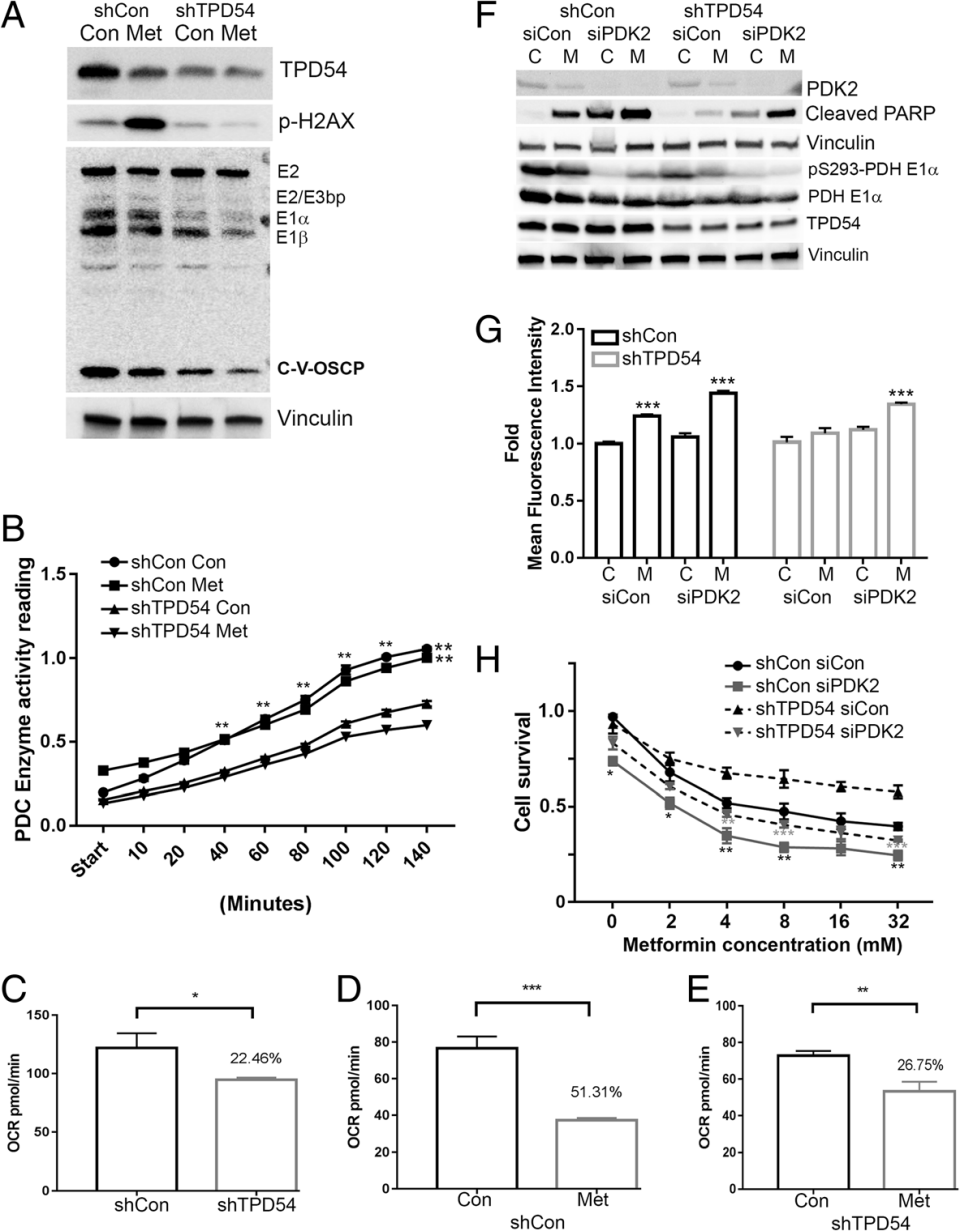
TPD54 is positively correlated with PDHE1 α protein and PDH complex enzyme activity, and through it changes the sensitivity of cells to metformin. **a** TPD54, p-H2AX, and PDH complex were detected by western blot in control and TPD54 stably knocked down cells treated with or without 8-mM metformin for 2 days. **b** PDH complex (PDC) enzyme activity was measured using cell lysates from cells described in **a**. shCon vs. shTPD54 with *p* < 0.01 **c–e** Oxygen consumption rate was measured using a Seahorse XFp analyzer for control and TPD54 stably knocked down cells (**c**), in control cells treated with or without 8-mM metformin (**d**), and in TPD54 stable knockdown cells treated with 1× PBS or 8 mM metformin (**e**). **f** Control and TPD54 stably knocked down cells transfected with either control siRNA or siPDK2 were treated with or without metformin for 2 days. Western blot assays for indicated proteins were performed. **g** Fold change of MitoSox mean fluorescence intensity was measured in control and TPD54 stably knocked down cells treated with 8-mM metformin for 2 days after knocking down PDK2.Sicon C vs. siCon M with *p* < 0.001, siPDK2 C vs. siPDK2 M with *p* < 0.001 in control cells. SiPDK2 C vs. siPDK2 M with *p* < 0.001 in TPD54 knocked down cells. **h** Cells were transfected with control, siPDK2, shTPD54 or siPDK2, and shTPD54 together**.** Cell survival was determined in the presence of the indicated metformin concentrations. shCon siCon vs. shCon siPDK2 or shTPD54 Con vs. shTPD54 siPDK2 were compared as indicated at different concentrations of metformin treatment. All data presented were representative experimental data that has been replicated by independent experiments. Data are reported as the mean ± SD from triplicate data of one experiment. Values are presented as mean ± SD, student *t* test was used to calculate *p* value for two-group comparisons with ***p < 0.001, **p < 0.01, *p < 0.05)

Next, we tested whether the association between TPD54 and PDH involved TPD54 regulation of PDH function, which could alter phenotypes associated with cellular metabolism and ROS production. Mitochondrial ROS production is tightly associated with active mitochondrial oxidative phosphorylation [35, 36]. Therefore, we hypothesized that the interaction between TPD54 and PDH might affect PDH activity and alter mitochondrial oxidative phosphorylation. Knocking down TPD54, decreased ROS production (Fig. 1e, f) was further confirmed by examining H2AX phosphorylation, a marker of DNA damage (Fig. 3a). Interestingly, we observed that the PDH complex, particularly E1 alpha, had a reduced protein level in TPD54 stably knocked down cells compared to controls (Fig. 3a), which was also correlated with reduced pyruvate dehydrogenase complex enzyme activity (Fig. 2b). The xenospheroid samples presented in Fig. 1g were blotted for PDH complex as well. The level of PDC complex showed reduced expression pattern when TPD54 levels were low across all the six samples (Additional file 5: Figure S3A). PDH is an important enzyme responsible for controlling oxygen consumption [37]. Consistent with the reduction in enzyme activity, there was also a 22.46% reduction in oxygen consumption rate (OCR) when TPD54 was knocked down (Fig. 3c). Metformin reduced OCR by 51.31% in control cells (Fig. 3d); while only 26.75% in TPD54 stably knockdown cells (Fig. 3e). We did not observe a significant difference in glycolysis either at the baseline or after metformin treatment between control and TPD54 knockdown cells (Additional file 7: Figure S3B, C and D). Metformin increased glycolysis in both control and TPD54 knockdown cells, and did not have effects on other non-glycolysis induced extracellular acidification. These data collectively demonstrate that TPD54 knockdown not only affects cellular oxygen consumption rate but also prevents metformin-mediated inhibition of OCR, which could be the outcome of its direct regulation of PDH protein levels and activity.

In order to answer these questions and to determine whether downregulation of TPD54 caused decreased cellular sensitivity to metformin as a direct result of PDH activity, PDH upstream inhibitory kinase, PDK2, the major kinase for serine 293 phosphorylation was knocked down in TPD54 stable knockdown cells. We found that PDH activity was partially recovered as demonstrated by reduced PDH E1α S293 phosphorylation (Fig. 3f). In control cells, metformin increased cleaved PARP and decreased live cells, effects which were further enhanced by downregulating PDK2 (Fig. 3g, h). In TPD54 knockdown cells, metformin’s effect was reduced as represented by decreased cleaved PARP and reduced cytotoxicity compared with control cells treated with metformin. Double knockdown of TPD54 and PDK2 compared with TPD54 single knockdown resulted in increased levels of cleaved PARP and ROS production, as well as reduced live cells in the presence of metformin (Fig. 3g, h). Furthermore, the activation of PDH by adding dichloroacetic acid (DCA) was performed as well. DCA showed similar effects as those observed with PDK2 knockdown, in that it restored cell sensitivity to metformin in TPD54 knockdown cells (Additional file 7: Figure S3E). On the other hand, when the cells were treated with CoCl_2_ to mimic the hypoxic conditions, metformin-induced cell death was totally abolished in both control and shTPD54 stable cells (Additional file 7: Figure S3F). These results demonstrated that metformin resistance caused by TPD54 knockdown was indeed the direct result of reduced PDH activity and reduced reactive oxidative species production.

### TPD54 knockdown leads to increased PDH E1α phosphorylation at serine 232 by PDK1 followed by increased ubiquitination and protein degradation

**Fig. 4:**
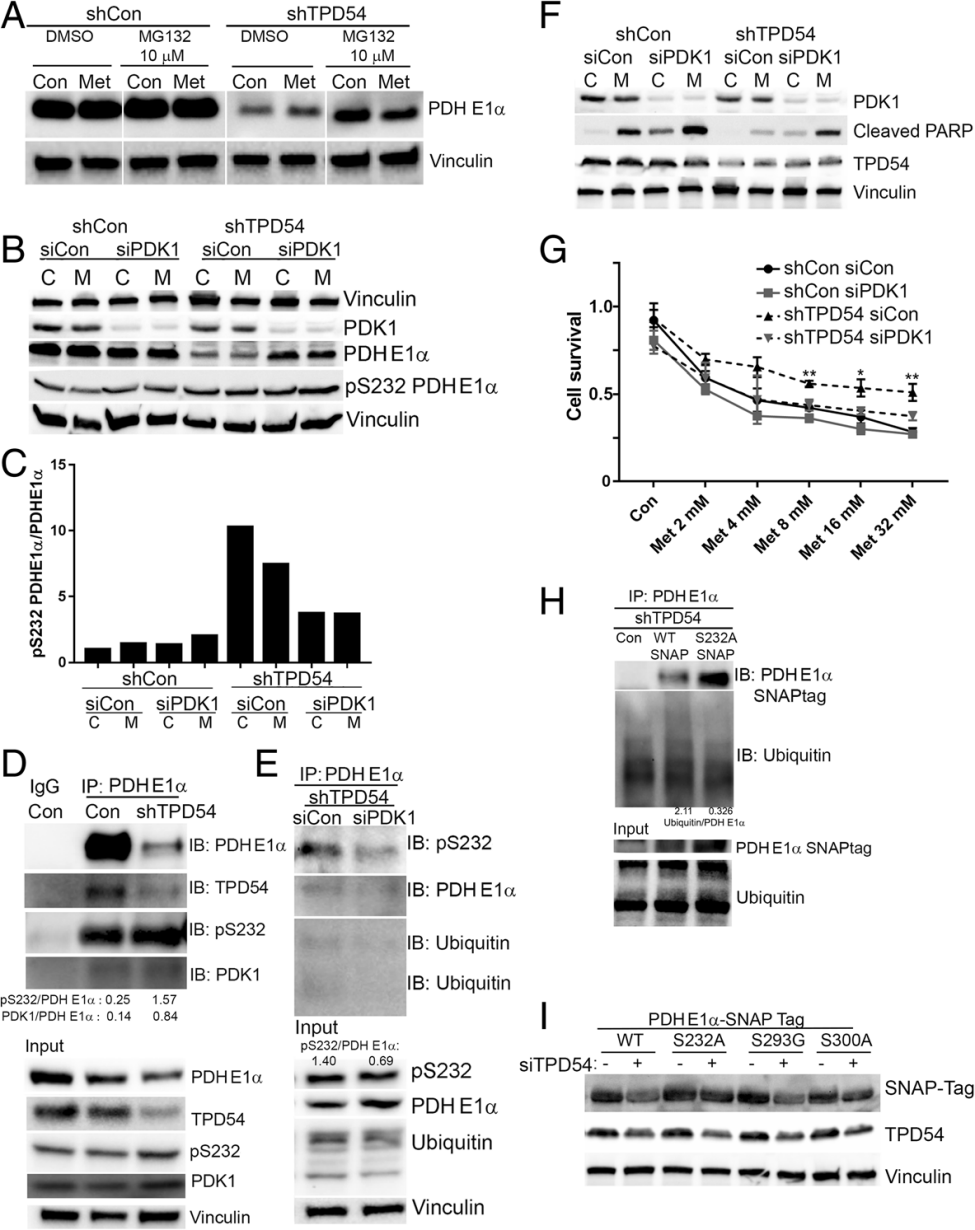
TPD54 knockdown leads to increased PDH E1α phosphorylation at serine 232 by PDK1 followed by increased ubiquitination and degradation. **a** PDH E1α protein levels were detected by western blot in control and TPD54 stably knocked down cells with either DMSO or MG132 (10 μM) treatment for 1 day. **b** control and TPD54 stably knocked down cells transfected with control siRNA or siPDK1 were treated with metformin for 2 days. Western blot assays were performed to detect indicated proteins. **c** The ratio of pS232 PDH E1α to total PDH E1α in Fig. 5b was quantified and plotted as a bar graph. **d** Immunoprecipitation (IP) of PDH E1α with lysate from control cell and TPD54 stably knocked down cells was performed followed by immunoblotting of indicated proteins. Input represents whole cell lysate. **e** immunoprecipitation (IP) of PDH E1α with lysate of TPD54 stably knocked down cells with or without transient PDK1 knockdown was performed followed by immunoblotting of indicated proteins. Total proteins were determined in the input. **f** Control and TPD54 stably knocked down cells were transiently transfected with control siRNA or SiPKD1, followed by metformin treatment for 2 days. Western blot was then performed to detect indicated proteins. **g** Cell survival was determined in cells transfected with control, siPDK1, shTPD54, or double knockdowns. Values are presented as mean ± SD, student *t* test was used to calculate *p* value for two-group comparisons at different concentrations of metformin treatment (shCon siCon vs. shCon siPDK1 or shTPD54 siCon vs. shTPD54 siPDK1 with ***p < 0.001, **p < 0.01, *p < 0.05). **h** Western blot was performed to detect transient transfected WT PDH E1α and PDH E1α S232A mutant using SNAP-tag antibody and its ubiquitination modification after immunoprecipitation PDH E1α in control cells or TPD54 stably knocked down cells. Ubiquitination status and PDH E1α in whole cell lysate was detected by western blot as demonstrated in Input. **i** Western blots detecting protein changes among wild-type PDH E1α and its mutants S232A, S293G, and S300A were performed using SNAP-tag antibody in MCF-7 cells with or without transient TPD54 knockdown. All data presented were representative experimental data that have been replicated by independent experiments

Considering the important role of PDH in cancer metabolic reprogramming, it is necessary to understand how TPD54 regulates PDH E1α protein level. We then examined the effects of TPD54 knockdown on PDH E1 α mRNA and protein degradation in MCF7 cells. TPD54 did not affect PDH E1α mRNA level (Additional file 8: Figure S4A); however, we found that the addition of proteasome inhibitor, MG132, compared to DMSO treatment, restored the decreased PDH E1α protein caused by TPD54 knockdown (Fig. 4a). This suggests that TPD54 is involved in regulating PDH E1α protein stability and its downregulation leads to increased PDH E1α protein degradation and, in turn, reduced enzyme activity. Han et al. [38] reported that EGFR-PTK-mediated tyrosine phosphorylation of the PDH E1β protein led to post-translational modification with enhanced ubiquitination and proteasome-mediated degradation of the PDH E1β subunit. It is likely that phosphorylation at individual PDH E1α sites might have similar effects on the regulation of protein stability as that discovered for PDH E1β.

There are four pyruvate dehydrogenases including PDK1, PDK2, PDK3, and PDK4 that can phosphorylate PDH E1α with varied specificities [39]. We have shown that the downregulation of PDK2 affects phosphorylation at serine site 293 and PDH enzyme activity (Fig. 3f). To determine which PDK isoforms might have an effect on PDH stability through phosphorylation, we downregulated PDK1, PDK2, PDK3, and PDK4 individually and examined their effects on PDH E1α protein levels. Interestingly, PDK1 knockdown specifically restored PDH E1α protein level in TPD54 knockdown cells (Fig. 4b). Since PDK1 mainly phosphorylates the serine 232 site, we determined the ratio between PDH E1α serine 232 phosphorylation and total PDH E1α in TPD54 knockdown cells. We found that TPD54 knockdown increased the phosphorylation of PDH E1α at serine 232, which was further reduced by downregulating PDK1 (Fig. 4c). To further demonstrate that TPD54 is involved in the regulation of PDH E1α phosphorylation at serine 232 and the potential mechanisms involved in this regulation, immunoprecipitation of PDH E1α was performed in TPD54 knockdown cells and showed increased PDK1 binding and increased PDH E1α phosphorylation at serine 232 relative to PDH E1α level compared with control cells (Fig. 4d), indicating that TPD54 might block PDK1 interaction with PDH E1α and, in turn, block the phosphorylation of PDH E1α. Since PDK1 is the only known kinase to phosphorylate PDH E1α at serine 232, PDK1 was transiently downregulated with siRNA in TPD54 stably knocked down cells (Fig. 4e). PDK1 knockdown increased the PDH E1α protein level and reduced the ratio of phosphorylated S232 PDH E1α to total PDH E1α (Fig. 4e). We observed reduced ubiquitination and serine 232 phosphorylation of PDH E1α when PDK1 was knockdown in PDH E1α immunoprecipitates (Fig. 4e). We also determined the PDK1 effect on metformin cytotoxicity. In control cells, metformin increased cleaved PARP and decreased live cells, an effect which were further enhanced by downregulating PDK1 (Fig. 4f, g). In TPD54 knockdown cells, metformin’s effect was reduced compared with control cells treated with metformin. Double knockdown of TPD54 and PDK1 compared with TPD54 knockdown alone resulted in increased levels of cleaved PARP and increased cytotoxicity in the presence of metformin (Fig. 4f, g). These results suggest that the loss of TPD54 leads to increased phosphorylation of PDH E1α at serine 232 by PDK1, resulting in increased PDH E1α protein degradation. PDK1 knockdown restores PDH E1α protein level by reducing serine 232 phosphorylation and subsequent ubiquitination of PDH E1α. To further elucidate the role of serine 232 phosphorylation in mediating PDH E1α ubiquitination and protein degradation by TPD54, PDH E1α wild type and PDH E1α S232A mutant were transfected into TPD54 stably knocked down cells. Immunoprecipitation of PDH E1α was performed to examine ubiquitination levels. SNAP-tag antibody was used to detect overexpressed PDH E1α wild type and S232A mutant. The PDH E1α S232A, which cannot be phosphorylated, has reduced ubiquitination compared to wild-type PDH E1α in TPD54 stably knocked down cells (Fig. 4h), suggesting that PDH E1α phosphorylation at serine 232 is required for ubiquitination. PDH E1α subunit is known to be phosphorylated at three different serine sites (S293, S300, and S232). Phosphorylation of PDH E1α at serine 293 by PDK2 is the most well-known mechanism for PDH E1α enzyme inactivation but there have been no reports of the involvement of additional phosphorylation sites in the regulation of protein stability. The role of phosphorylation at PDH E1α serine 232 and serine 300 are not well understood [28]. To date, PDH E1α serine 232 is known to be phosphorylated only by PDK1. PDH E1α serine 300 can be specifically phosphorylated by PDK4, followed by PDK1, 2, and 3. Since serine 300 can also be phosphorylated by PDK1, we also tested whether this site might be involved in PDH E1α degradation. In order to test this possibility, site-directed mutagenesis was performed to create PDH E1α mutants at all three known serine phosphorylation sites to examine the effects of TPD54 knockdown on PDH E1α stability. We overexpressed PDH E1α wild type and three different PDH E1α mutants, S232A, S293G, and S300A fused with SNAP-tag in MCF-7 cells. We then examined the protein changes of transfected PDH E1α using SNAP-tag antibody after transiently knocking down TPD54. Transient TPD54 knockdown reduced PDH E1α wild type and S293G mutant. The downregulation of TPD54 did not significantly reduce protein levels of S232A and S300A mutants when compared with the WT and S293G mutants (Fig. 4i). In summary, PDK1 phosphorylation of PDH E1α at serine 232 and 300 sites is important for further Ub-dependent degradation of PDH E1α. TPD54 prevents PDH E1α degradation by preventing PDK1 phosphorylation of PDH E1α 232 and 300 serine sites.

### Proposed model of the mechanisms of TPD54 in regulation of PDH E1α in breast cancer cells and its effect on metformin response

**Fig. 5:**
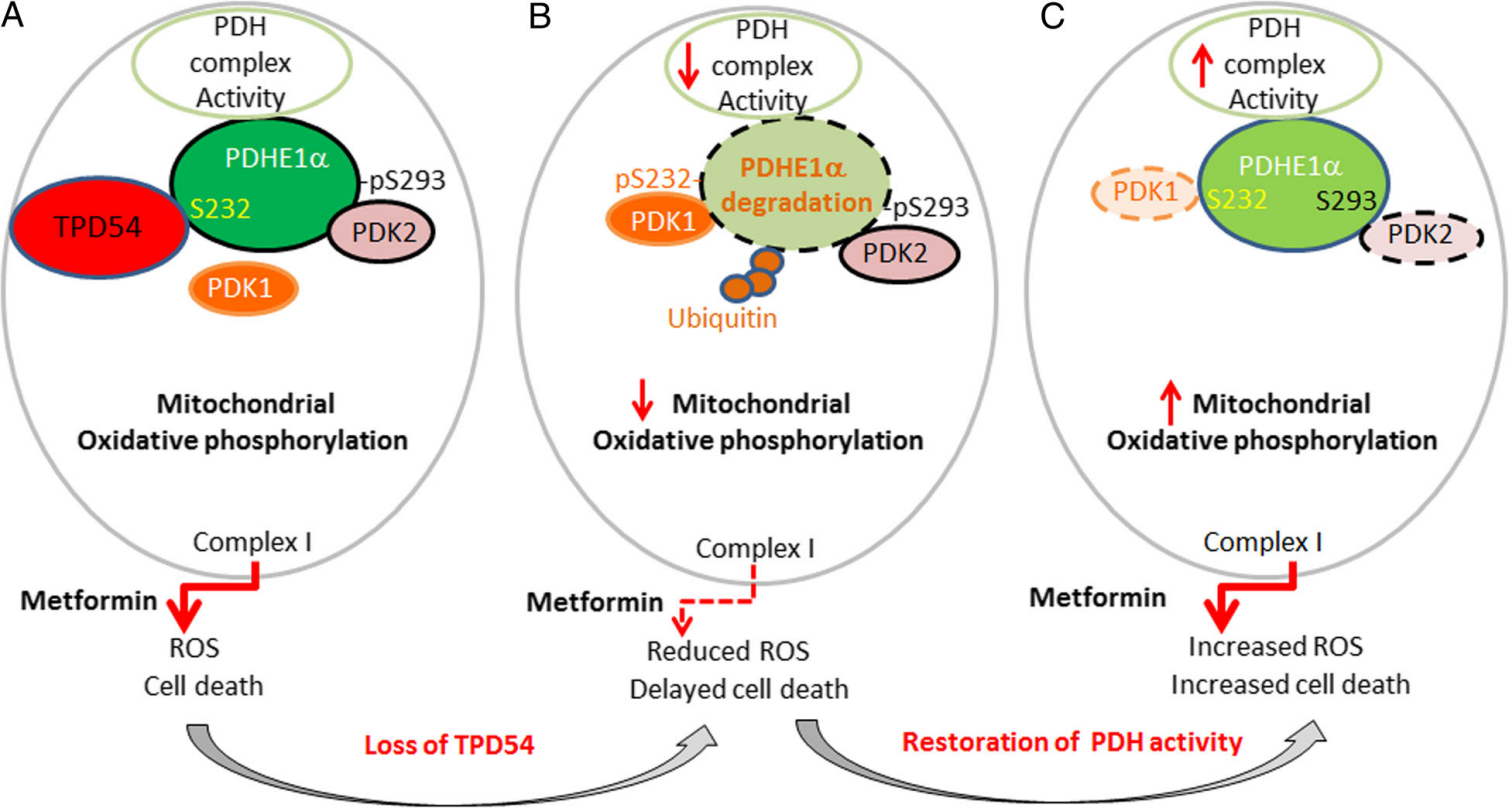
Proposed model of the mechanisms of TPD54 in regulation of PDH E1α in breast cancer cells and its effect on metformin response. TPD54 interacts with pyruvate dehydrogenase complex and affects PDH protein and enzyme activity. Loss of TPD54 causes increased phosphorylation of PDH E1α at S232 by PDK1, resulting in further ubiquitination and protein degradation of PDH E1α, and this phenotype can be restored by PDK1 knockdown (**b**, **c**). Loss of TPD54 leads to reduced oxidative phosphorylation and ROS production, which contribute to reduced metformin cytotoxicity (**a**, **b**). This can be reversed by activating pyruvate dehydrogenase by knocking down upstream inhibitory kinases PDK2 (**b**, **c**)

In this study, we have demonstrated that the TPD54 interacts with pyruvate dehydrogenase complex and affects PDH protein and enzyme activity. Deletion of TPD54 facilitates the phosphorylation of PDH E1α by PDK1, resulting in increased phosphorylation of PDH E1α at S232 and S300 and further ubiquitination and protein degradation of PDH E1α (Fig. 5b). This degradation can be prevented by PDK1 knockdown (Fig. 5c). This newly discovered function of TPD54 in the regulation of PDH further emphasizes the importance to have enhanced activity of PDH and oxidative phosphorylation in order for metformin to induce ROS production and cell death. The loss of TPD54 leads to reduced oxidative phosphorylation and ROS production, which contributes to reduced metformin cytotoxicity (Fig. 5a, b). However, this can be reversed by activating pyruvate dehydrogenase by knocking down PDK2 (Fig. 5b, c).

## Discussion

Previously identified proteins that interact with TPD54 include TPD52 [40], MAL proteolipid family (MAL2) [41], and phosphatase of regenerating liver (PRL1) using a yeast two-hybrid system [42]. The interaction with TPD52 and MAL2 potentially implicates TPD54’s role in cell proliferation and vesicle transport, respectively. The PRL-1 binding proteins identified by Lee et al. included TPD54, NDUFB8 (NADH:ubiquinone oxidoreductase subunit B8), and FKBP38. NDUFB8 and FKBP38 are known to have mitochondrial localizations [43]. Now, we have shown that TPD54 also has a mitochondrial distribution and interacts with PDH E1α in breast cancer cells, indicating its potentially important role in the metabolic regulation in cancer.

Pyruvate dehydrogenase (PDH) is a key rate-limiting enzyme that determines the metabolic balance between glycolysis versus mitochondrial oxidative phosphorylation [44]. It can be phosphorylated and inactivated by pyruvate dehydrogenase kinases (PDKs). This leads to the inhibition of mitochondrial oxidative phosphorylation and enhanced aerobic glycolysis, a hallmark of metabolic reprogramming for tumor growth and resistance to therapy [45]. Our data demonstrates that the protein interaction between TPD54 and PDH E1α affects PDH E1α protein stability and cell metabolism. TPD54 knockdown reduces PDH E1α protein and alters cellular metabolism by decreasing mitochondrial oxidative phosphorylation and increasing aerobic glycolysis. PDH E1α degradation in TPD54 knockdown cells could be partially restored by the proteasome inhibitor MG132. This indicates that TPD54 plays an important role in regulating PDH E1α protein stability. Once the enzyme activity of PDH E 1α is enhanced in TPD54 knockdown cells by knockdown of upstream inhibitory kinases, PDK2 or using PDK inhibitor such as DCA, despite reduced PDH E1α protein level, cellular sensitivity to metformin treatment can be restored to levels similar to that of control cells. These data collectively reveal a new function of TPD54 in altering cellular metabolism and response to metformin by regulating PDH E1α. Metformin-induced cell death was totally abolished when CoCl_2_ were added to mimic the hypoxic condition. This also emphasizes the importance of mitochondrial oxidative phosphorylation in determining cellular response to metformin, which provides valuable information for better individualizing metformin treatment. TPD54 knockdown reduced PDH activity and oxygen consumption; this is similar to the effects of CoCl_2_ and hypoxia. This could be an important factor to consider when treating tumors in vivo with metformin where some tumors are likely to have hypoxic microenvironment. Reducing hypoxia might be a reasonable strategy to enhance metformin cytotoxicity for cancer treatment in vivo.

The effects of TPD54 on metformin sensitivity were also observed with the other two complex I inhibitors (piericidin A and rotenone), which suggested an important role of TPD54 in regulating mitochondrial complex I function. Therefore, the complex I OCR was measured with complex I specific substrates in control and TPD54 knocked down cells with or without metformin. Metformin inhibited complex I in both control and TPD54 knocked down cells (Additional file 8: Figure S4B, C). The inhibitory effect of TPD54 knockdown on complex I OCR was seen for all substrates except the high concentration of pyruvate (10 mM) (Additional file 8: Figure S4B, 4C). This could be due to the fact that pyruvate induced reactivation of PDH in TPD54 knocked down cells. The inhibition of complex I was anticipated to affect NAD/NADH ratio [46]. Our results demonstrated a significant reduction in NAD+, NAD/NADH ratio, and NADH accumulation after metformin treatment in both control and TPD54 knocked down cells (Additional file 8: Figure S4D-F). The reduction of NAD level by metformin was more prominent in control cells than in shTPD54 stable cells. As shown in Fig. 1e, f and Fig. 2a, a significantly higher ROS was produced in control cells upon metformin treatment. The activation of PARP by ROS-induced DNA damage could contribute to a more rapid depletion of NAD+ in control cells than in shTPD54 cells (Additional file 8: Figure S4D). The addition of NAD+ or nicotinamide as extraneous resource of NAD+ could prevent the cell death induced by metformin in both control and shTPD54 cells (Additional file 8: Figure S4G and H). Future studies will be necessary to further elucidate how TPD54 might regulate mitochondrial function and, in turn, respond to complex I inhibitors.

Current understanding of PDH E1α protein stability is derived from studying the disease pyruvate dehydrogenase (PDH)-complex deficiency, an inborn error of metabolism that most commonly occurs due to mutations in the E1α subunit gene (PDHA1). To date, more than 90 mutations of the PDH complex have been identified. Several studies have demonstrated that certain mutations within the PDHA1 gene (E1α subunit) can result in decreased protein stability, reduced protein levels, and PDH enzyme activity. Besides genetic mutations, Han et al. [38] have demonstrated that the EGFR-PTK-mediated tyrosine phosphorylation of the E1β protein leads to post-translational modifications involving increased ubiquitination and proteasome-mediated degradation of the PDH E1β subunit. Here, we have demonstrated in breast cancer cells that PDH E1α protein levels are regulated by TPD54 protein through its interaction with PDH E1α, blocking upstream kinases, PDKs, and phosphorylating specific sites on PDH E1α, including 293 and possibly 300, resulting in increased ubiquitination and protein degradation. This finding could enhance our understanding of patients with specific symptoms related to PDH complex deficiency but who lack known genetic mutations.

There are four pyruvate dehydrogenases including PDK1, PDK2, PDK3, and PDK4, all of which can phosphorylate PDH E1α with different specificities [39]. The four pyruvate dehydrogenase kinases (PDK1, PDK2, PDK3, and PDK4) have varied catalytic activity toward PDH E1α. PDK2 possesses the greatest activity to phosphorylate PDH E1α at serine 293, the major site known to influence enzyme activity, followed by PDK4, PDK1, and PDK3. PDK4 phosphorylates PDH E1α at serine 300 with higher affinity than PDK1, 2, and 3. PDK1 is the only kinase known to phosphorylate PDH E1α at serine 232, but its mechanism of PDH E1α enzyme inactivation is not well understood. In our study, we demonstrate that TPD54 is important in regulating PDH E1α protein stability by preventing PDH E1α phosphorylation at serine 232 and serine 300 by PDK1 (Fig. 4). Therefore, in addition to its known mechanism of regulating enzyme activity by post-translational modification, in this case, phosphorylation also contributes significantly to PDH E1α function by increasing PDH E1α ubiquitination and degradation. During this process, TPD54 plays a major role by blocking access of upstream kinases and preventing phosphorylation of PDH E1α, affecting PDH E1α stability in breast cancer cells. Obviously, we cannot exclude the possibility that other potential modifications beyond phosphorylation might also contribute to this process.

It has been suggested that TPD54 is a tumor-related protein. Based on our results, TPD54 stabilizes PDH E1α and its overexpression in breast cancer cells might be a compensatory feedback mechanism to maintain the status of mitochondrial oxidative phosphorylation. Studies are limited with regard to the biological function of TPD54 in cancer cells, especially in breast cancer. In oral squamous cell carcinoma cells, TPD54 was reported to be a negative regulator of tumor progression by downregulating anchorage-independent cell growth and migration in vitro and by attenuating tumor growth in vivo [12]. Our data demonstrate that, in breast cancer cells, downregulation of TPD54 can reduce cellular oxygen consumption, increase colony formation, and decrease cancer cell sensitivity to metformin treatment. Overexpression of TPD54 inhibits colony formation and enhances metformin cytotoxicity in breast cancer cells. These findings suggest that TPD54 acts as a negative regulator of cell growth in breast cancer as observed in oral squamous carcinoma cells. We understand that even though our in vitro finding indicated TPD54 as a tumor suppressor, however, its regulation on PDH and cancer metabolism might have different effects on tumorigenesis and cancer proliferation or metastasis in vivo*.* It has been shown that in vivo, cancer cells show more oxidative phosphorylation than those same cells in culture and that oxidative phosphorylation is advantageous for growth and metastasis in vivo [47, 48]. This necessitates future in vivo experiments to be performed in order to further define the function of TPD54 in breast cancer and its role in drug treatment response.

## Conclusions

TPD54 has important biological functions in breast cancer. It reduces breast cancer proliferation and enhances cellular sensitivity to metformin treatment in breast cancer cell lines and in breast cancer patient-derived xenografts by modulating the PDH enzyme activity in vitro. It prevents PDK1-mediated phosphorylation of PDHE1α at serine 232 and S300 and, therefore, reduces PDH E1α ubiquitination and protein degradation. On the other hand, TPD54 downregulation increases PDH E1α protein degradation and leads to decreased PDH enzyme activity, which reduces mitochondrial oxygen consumption and reactive oxygen species (ROS) production, thus contributing to the resistance of breast cancer cells to metformin treatment. These findings provide new insights into understanding the role of TPD54 in the regulation of PDH complex, cancer metabolic reprogramming, and the mechanisms of metformin resistance in breast cancer with low TPD54 expression. These further highlight its potential application as a biomarker for selecting tumors that may be sensitive to metformin treatment.

## Additional files

Additional file 1: Supplementary Materials and Methods. (DOCX 18 kb)

Additional file 2: **Figure S2.** A. Fold changes of cell number was measured and plotted for shCon and shTPD54 stable cells. B. Fold change of cell number was measured and plotted for vector control cells and cells overexpressing TPD54. Data were reported as the mean ± SD from triplicate data of one experiment. Values are presented as mean ± SD, student t-test was used to calculate *p* value for two group comparisons with ***p < 0.001, **p < 0.01 and *p < 0.05). All experiments were repeated independently for at least one time. C. 8-bit grayscale of individual channels of dsMito and TPD54 in MCF7, BT549, MDAMB231 and T47D was presented. The overlap coefficient of signals from the two individual channels was 0.87, 0.96, 0.96 and 0.88 respectively. D. 8-bit grayscale of individual channels of PDH E1alpha and TPD54 in MCF7 and MDAMB231was presented and the overlap coefficient of signals from the two individual channels was 0.98 and 0.96, respectively. (TIF 7743 kb)

Additional file 3: Primers used in preparing PDHE1α mutants S232A, S300A and S293G, and the sequencing results. (XLSX 14 kb)

Additional file 4: **Figure S1.** A. MTS assay was performed and plotted for the cell proliferation of shcon and shTPD54 cells with or without 2 mM metformin. B. mCherry vector and mCherry-TPD54 were transfected into shcon and shTPD54 stable cells for 2 days. Western blot detected the expression of mCherry and mCherry-TPD54. C. mCherry vector and mCHerry-TPD54 were transfected into shcon and shTPD54 stable cells for 1 day and treated with metformin (8 mM) for 2 days. Percentage of dead cells was plotted. D. shTPD54 virus particles purchased from Santa Cruz was used to infect MCF7 cells, western blot detected TPD54 level. E. Percentage of cell death (%) was plotted after treatment with different concentrations of metformin for 4 days in cells infected with either control virus or shTPD54 virus particles. F. G. Shcon and shTPD54 cells were treated with Piericidin A or rotenone at indicated concentration for 2 days, the percentage of cell death were measured using Sytox green assay. Data were reported as the mean ± SD from triplicate data of one experiment. Values are presented as mean ± SD, student t-test was used to calculate *p* value for two group comparisons with ****p < 0.0001 and ***p < 0.001, **p < 0.001 and *p < 0.05). All experiments were repeated independently for at least one time. (TIF 23150 kb)

Additional file 5: Supplementary Figure legends. (DOCX 17 kb)

Additional file 6: Mass spectrometry results of proteins coimmunoprecipitated with TPD54 in MCF7 cells. (XLSX 51 kb)

Additional file 7: **Figure S3.** A. Western blot detected PDH complex using the same samples presented in Fig. 1g. B. ECAR levels in shcon and shTPD54 stable cells were measured. C. ECAR levels in shCon cells were measured after treated with metformin (8 mM) for 1 h. D. ECAR levels in shTPD54 cells were measured after treated with metformin (8 mM) for 1 h. E. Percentage of dead cells (%) was plotted for shcon and shTPD54 cells treated with different concentrations of metformin with or without DCA (5 mM) for 3 days. Shcon con vs. shCon DCA, and shTPD54 con vs. shTPD54 DCA were compared at different concentration of metformin treatment. F. The percentage of dead cells (%) was plotted after treated with metformin at different concentrations in the addition of CoCl_2_ for 3 days. Shcon con vs. shCon CoCl2, and shTPD54 con vs. shTPD54 CoCl2 were compared at different concentration of metformin treatment. Data were reported as the mean ± SD from triplicate data of one experiment. Values are presented as mean ± SD, student t-test was used to calculate *p* value for two group comparisons with ****p < 0.0001, ***p < 0.001 and **p < 0.01). All experiments were repeated independently for at least one time. (TIF 24927 kb)

Additional file 8: **Figure S4.** A. qRT-PCR results for mRNA levels of actin, AMPK, PDH E2, PDH E3bp and PDH E1α from MCF7 control and TPD54 stably knocked down cells. B. Complex I OCR were measured in the present of pyruvate (10 mM) at base line and after metformin treatment in control and TPD54 knocked down cells. C. Complex I OCR were measured in the absent of pyruvate at base line or after metformin treatment in control and TPD54 knocked down cells. D. E. F. NAD+, NADH, and NAD+/NADH ratio were measured in shCon and shTPD54 cells with or without metformin (8 mM) for 1.5 days respectively. G. Percentage of dead cells (%) was plotted for shcon and shTPD54 cells treated with different concentrations of metformin with or without NAD+ (1.5 mM) for 3 days. Shcon con vs. shcon NAD, and shTPD54 con vs. shTPD54 NAD were compared. H. The percentage of dead cells (%) was plotted after treated with metformin at different concentrations in the addition of nicotinamide (5 mM) for 3 days. Shcon con vs. shcon nicotinamide, and shTPD54 con vs. shTPD54 nicotinamide were compared at different concentrations of metformin treatment. Values are presented as mean ± SD, student t-test was used to calculate *p* value for two group comparisons with ****p < 0.0001, ***p < 0.001 and **p < 0.01). All experiments were repeated independently for at least one time. (TIF 25370 kb)

## Abbreviations

LCL: Lymphoblastoid cell lines
PDH: Pyruvate dehydrogenase
PDK: Pyruvate dehydrogenase kinase
ROS: Mitochondrial reactive oxygen species
TPD54: Tumor protein D54
