# Supplementary material for: The novel function of tumor protein D54 in regulating pyruvate dehydrogenase and metformin cytotoxicity in breast cancer
Source: Cancer Metab. 2019 Jan 24;7:1. doi: 10.1186/s40170-018-0193-4 (PMC6345044; doi:10.1186/s40170-018-0193-4)
Supplement: Supplementary file 1 — Supplementary Materials and Methods. (DOCX 18 kb) [file 40170_2018_193_MOESM1_ESM.docx]

**Supplementary Materials and Methods**

**siRNA and shRNA information**

**ShTPD54 plasmids (TRC2-pLKO-puro, TPD52L2, NM_003288) were purchased from Sigma.** NM_003288.2-1014s21c1 Target sequence: GCGGAGGGTTTGAAAGAATAT, NM_003288.2-1692s21c1 Target sequence: CCCTGTCTTTAGCACCCTTTA, NM_003288.2-517s21c1 Target sequence: CAGGAAACTCTTTCACAGGCA, NM_003288.2-592s21c1 Target Sequence: CTTGGAGACATGAGGAACTCT, NM_003288.2-645s21c1 Target Sequence: GACCATAAAGTCTAAGGTTGT.

**SiRNAs targeting TPD54, PDK2 were purchased from Dharmacon, detailed sequence information were listed below:**

| **Pool Catalog Number** | **Duplex Catalog Number** | **Gene Symbol** | **GENE ID** | **Gene Accession** | **GI Number** | **Sequence** |
| --- | --- | --- | --- | --- | --- | --- |
| M-019568-01 | D-019568-01 | TPD52L2 | 7165 | NM_199359 | 40805861 | GGGCUGAGCUUACCAAGGU |
| M-019568-01 | D-019568-03 | TPD52L2 | 7165 | NM_199359 | 40805861 | CCUGAAUUCUCCUAACAAA |
| M-019568-01 | D-019568-04 | TPD52L2 | 7165 | NM_199359 | 40805861 | GGAUGUUCCUGUCGACACA |
| M-019568-01 | D-019568-17 | TPD52L2 | 7165 | NM_199359 | 40805861 | GAAUCUAGCUUUAACGGAA |
| M-005020-00 | D-005020-01 | PDK2 | 5164 | NM_002611 | 31543388 | CAAAGAUGCCUACGACAUG |
| M-005020-00 | D-005020-02 | PDK2 | 5164 | NM_002611 | 31543388 | GCACGGAGCCCAAGAACAC |
| M-005020-00 | D-005020-03 | PDK2 | 5164 | NM_002611 | 31543388 | GCUCCUGUGUGACAAGUAU |
| M-005020-00 | D-005020-04 | PDK2 | 5164 | NM_002611 | 31543388 | CCAGCACACCCUCAUCUUU |

**D54 shRNA (h) Lentiviral Particles (sc-77088-V) is a pool of 3 different shRNA plasmids and purchased from Santa Cruz.**

sc-77088-VA: Hairpin sequence: GATCCGCGTTTGCATGAATTTGAATTCAAGAGATTCAAATTCATGCAAACGCTTTTT. Corresponding siRNA sequences (sc-77088A), sense: GCGUUUGCAUGAAUUUGAAtt, antisense: UUCAAAUUCAUGCAAACGCtt;

sc-77088-VB: Hairpin sequence: GATCCGCTTTAGCCTCATAGAATATTCAAGAGATATTCTATGAGGCTAAAGCTTTTT. Corresponding siRNA sequences (sc-77088B), sense: GCUUUAGCCUCAUAGAAUAtt, antisense: UAUUCUAUGAGGCUAAAGCtt

sc-77088-VC: Hairpin sequence: GATCCGGATTGCCGTTACAGTCTTTTCAAGAGAAAGACTGTAACGGCAATCCTTTTT Corresponding siRNA sequences (sc-77088C),sense: GGAUUGCCGUUACAGUCUUtt, antisense: AAGACUGUAACGGCAAUCCtt.

All sequences are provided in 5′ → 3′ orientation.

**PDK1 siRNA (sc-36203) was purchased from Santa Cruz**.

sc-36203A: sense: CGAGGAGACAGAAACUGAAtt, antisense: UUCAGUUUCUGUCUCCUCGtt; sc-36203B: sense: GAGACUGUGUUGUUAGUUAtt, antisense: UAACUAACAACACAGUCUCtt; sc-36203C: sense: GUAGAGUUUGUAUGUUUGAtt, antisense: UCAAACAUACAAACUCUACtt. All sequences are provided in 5′ → 3′ orientation.

**Silver staining**

Proteins were separated on Biorad precast polyacrylamide gel (Cat#4561084). Gels were stained with the Silver Stain Kit for Mass Spectrometry (Thermo Scientific Pierce, PI24600) following the vendor’s protocol. Protein bands were cut with a sterilized blade and detained with the same kit. Samples were sent to the Taplin Mass Spectrometry facility at Harvard Medical School for analysis of potential interacting proteins.

**NAD+/NADH Quantitation Colorimetric Kit**

The kit was purchased from Biovision (K337). Cells were treated with or without metformin (8 mM) for 1.5 days. Cells were washed with 1X PBS and trypsinized to obtain the total cell numbers. Cell pellets with same cell number were lysed with the provided extraction buffer. Total NAD+ level was directly measured with the enzyme mix, for NADH measurement, cell lysate was incubated at 60°C for 30 minutes which lead to the degradation of NAD+. NAD+ was calculated by subtracting NADH from the total measured NAD+.

**Complex I OCR assay**

Cells were seeded at 10,000 cells per well and incubated overnight. Media in the wells was switch to mitochondrial assay buffer supplemented with 10 mM ADP.

Complex I OCR with pyruvate was performed with media containing the respiration substrates of complex I (10 mM pyruvate, 1 mM malic acid, Palmitoyl carnitine (50 μM), glutamine (1 mM), and PMP (1nM) was injected from Port A, metformin (8 mM) was injected from Port B. Complex I OCR without pyruvate was performed with media containing the respiration substrates of complex I (1 mM malic acid, Palmitoyl carnitine (100 μM), glutamine (2 mM), PMP (1nM) was injected from Port A, metformin (8 mM) was injected from Port B. Media containing oligomycin (1μM) were injected sequentially from Port C. Complex I OCR was calculated after the OCR reading was stabilized after injection from Port A. The effect of metformin on OCR was measured after the injection from Port B.
