# Supplementary material for: The novel function of tumor protein D54 in regulating pyruvate dehydrogenase and metformin cytotoxicity in breast cancer
Source: Cancer Metab. 2019 Jan 24;7:1. doi: 10.1186/s40170-018-0193-4 (PMC6345044; doi:10.1186/s40170-018-0193-4)
Supplement: Supplementary file 5 — Supplementary Figure legends. (DOCX 17 kb) [file 40170_2018_193_MOESM5_ESM.docx]

**Supplementary Figure 1: A.** MTS assay was performed and plotted for the cell proliferation of shcon and shTPD54 cells with or without 2 mM metformin. **B.** mCherry vector and mCherry-TPD54 were transfected into shcon and shTPD54 stable cells for two days. Western blot detected the expression of mCherry and mCherry-TPD54. **C.** mCherry vector and mCHerry-TPD54 were transfected into shcon and shTPD54 stable cells for one day and treated with metformin (8 mM) for two days. Percentage of dead cells was plotted. **D.** shTPD54 virus particles purchased from Santa Cruz was used to infect MCF7 cells, western blot detected TPD54 level. **E.** Percentage of cell death (%) was plotted after treatment with different concentrations of metformin for 4 days in cells infected with either control virus or shTPD54 virus particles. **F. G.** Shcon and shTPD54 cells were treated with Piericidin A or rotenone at indicated concentration for 2 days, the percentage of cell death were measured using Sytox green assay. Data were reported as the mean ± SD from triplicate data of one experiment. Values are presented as mean ± SD, student t-test was used to calculate p value for two group comparisons with ****p < 0.0001 and ***p < 0.001, **p < 0.001 and *p < 0.05). All experiments were repeated independently for at least one time.

**Supplementary Figure 2: A.** Fold changes of cell number was measured and plotted for shCon and shTPD54 stable cells. **B.** Fold change of cell number was measured and plotted for vector control cells and cells overexpressing TPD54. Data were reported as the mean ± SD from triplicate data of one experiment. Values are presented as mean ± SD, student t-test was used to calculate p value for two group comparisons with ***p < 0.001, **p < 0.01 and *p < 0.05). All experiments were repeated independently for at least one time. **C.** 8-bit grayscale of individual channels of dsMito and TPD54 in MCF7, BT549, MDAMB231 and T47D was presented. The overlap coefficient of signals from the two individual channels was 0.87, 0.96, 0.96 and 0.88 respectively. **D.** 8-bit grayscale of individual channels of PDH E1alpha and TPD54 in MCF7 and MDAMB231was presented and the overlap coefficient of signals from the two individual channels was 0.98 and 0.96, respectively.

**Supplementary Figure 3: A.** Western blot detected PDH complex using the same samples presented in Figure 1G. **B.** ECAR levels in shcon and shTPD54 stable cells were measured. **C.** ECAR levels in shCon cells were measured after treated with metformin (8mM) for one hour. **D.** ECAR levels in shTPD54 cells were measured after treated with metformin (8mM) for one hour. **E.** Percentage of dead cells (%) was plotted for shcon and shTPD54 cells treated with different concentrations of metformin with or without DCA (5 mM) for 3 days. shcon con vs. shCon DCA, and shTPD54 con vs. shTPD54 DCA were compared at different concentration of metformin treatment. **F.** The percentage of dead cells (%) was plotted after treated with metformin at different concentrations in the addition of CoCl2 for 3 days. shcon con vs. shCon CoCl2, and shTPD54 con vs. shTPD54 CoCl2 were compared at different concentration of metformin treatment. Data were reported as the mean ± SD from triplicate data of one experiment. Values are presented as mean ± SD, student t-test was used to calculate p value for two group comparisons with ****p < 0.0001, ***p < 0.001 and **p < 0.01). All experiments were repeated independently for at least one time.

**Supplementary Figure 4: A.** qRT-PCR results for mRNA levels of actin, AMPK, PDH E2, PDH E3bp and PDH E1α from MCF7 control and TPD54 stably knocked down cells. **B.** Complex I OCR were measured in the present of pyruvate (10 mM) at base line and after metformin treatment in control and TPD54 knocked down cells. **C.** Complex I OCR were measured in the absent of pyruvate at base line or after metformin treatment in control and TPD54 knocked down cells. **D. E. F.** NAD+, NADH, and NAD+/NADH ratio were measured in shCon and shTPD54 cells with or without metformin (8mM) for 1.5 days respectively. **G.** Percentage of dead cells (%) was plotted for shcon and shTPD54 cells treated with different concentrations of metformin with or without NAD+ (1.5 mM) for 3 days. shcon con vs. shcon NAD, and shTPD54 con vs. shTPD54 NAD were compared. **H.** The percentage of dead cells (%) was plotted after treated with metformin at different concentrations in the addition of nicotinamide (5 mM) for 3 days. shcon con vs. shcon nicotinamide, and shTPD54 con vs. shTPD54 nicotinamide were compared at different concentrations of metformin treatment. Values are presented as mean ± SD, student t-test was used to calculate p value for two group comparisons with ****p < 0.0001, ***p < 0.001 and **p < 0.01). All experiments were repeated independently for at least one time.

**Supplementary file 1:** Mass spectrometry results of proteins coimmunoprecipitated with TPD54 in MCF7 cells.

**Supplementary file 2:** Primers used in preparing PDHE1α mutants S232A, S300A and S293G, and the sequencing results.
